# The Development and Evaluation of Reagentless Glucose Biosensors Using Dendritic Gold Nanostructures as a Promising Sensing Platform

**DOI:** 10.3390/bios13070727

**Published:** 2023-07-13

**Authors:** Natalija German, Anton Popov, Almira Ramanaviciene

**Affiliations:** 1 Department of Immunology, State Research Institute Centre for Innovative Medicine, LT-08406 Vilnius, Lithuania; natalija.german@imcentras.lt (N.G.); anton.popov@chgf.vu.lt (A.P.); 2 NanoTechnas—Center of Nanotechnology and Materials Science, Institute of Chemistry, Faculty of Chemistry and Geosciences, Vilnius University, LT-03225 Vilnius, Lithuania

**Keywords:** constant potential amperometry, ferrocenecarboxylic acid, glucose oxidase, gold nanostructures, graphite rod electrode, polymerization, phenanthroline-dione, tetramethylbenzidine, tetrathiafulvalene

## Abstract

Reagentless electrochemical glucose biosensors were developed and investigated. A graphite rod (GR) electrode modified with electrochemically synthesized dendritic gold nanostructures (DGNs) and redox mediators (Med) such as ferrocenecarboxylic acid (FCA), 1,10-phenathroline-5,6-dione (PD), N,N,N′,N′-tetramethylbenzidine (TMB) or tetrathiafulvalene (TTF) in combination with glucose oxidase (GOx) (GR/DGNs/FCA/GOx, GR/DGNs/PD/GOx, GR/DGNs/TMB/GOx, or GR/DGNs/TTF/GOx) were developed and electrochemically investigated. A biosensor based on threefold-layer-by-layer-deposited PD and GOx (GR/DGNs/(PD/GOx)_3_) was found to be the most suitable for the determination of glucose. To improve the performance of the developed biosensor, the surface of the GR/DGNs/(PD/GOx)_3_ electrode was modified with polypyrrole (Ppy) for 5 h. A glucose biosensor based on a GR/DGNs/(PD/GOx)_3_/Ppy_(5 h)_ electrode was characterized using a wide linear dynamic range of up to 39.0 mmol L^−1^ of glucose, sensitivity of 3.03 µA mM^−1^ cm^−2^, limit of detection of 0.683 mmol L^−1^, and repeatability of 9.03% for a 29.4 mmol L^−1^ glucose concentration. The Ppy-based glucose biosensor was characterized by a good storage stability (*τ*_1/2_ = 9.0 days). Additionally, the performance of the developed biosensor in blood serum was investigated.

## 1. Introduction

Interest in accurate, inexpensive, sensitive, selective, miniature, and rapid electrochemical biosensors [1,2,3,4,5] for clinical and pharmaceutical chemistry, drug discovery, and food and environmental quality monitoring has increased in the last few decades [1,2,3,4,5,6,7]. Glucose, as a monosaccharide, has significant importance as a vital energy source for organisms [2,7]. It is estimated that the number of deaths from diabetes in 2030 will account for 3.5% of deaths caused by non-infectious diseases [8]. The concentration of glucose in the bloodstream serves as the primary indicator for diagnosing and monitoring patients with diabetes mellitus [1,3]. Because of the high concentration of glucose in the blood of diabetic patients, the retina, kidneys, and the nervous and circulatory systems are damaged [3]. More than USD 13 billion and approximately 85% of the global biosensor market are used for the medical diagnostics of glucose to treat and prevent this disease [3,8].

The performance of glucose biosensors, as well as their longer operational stability and sensitivity, depends on many factors, including proper glucose oxidase (GOx) immobilization. For this purpose, the adsorption of GOx on the working electrode surface is ineffective in most cases; thus, additional crosslinking of GOx, covalent binding to a pre-modified surface or encapsulation in polymers is required [4,5,9]. Additionally, nanomaterials present on the electrode surface can improve the proper spatial orientation and distribution of GOx molecules and ensure higher catalytic activity. Nanomaterials such as carbon nanotubes (CNTs), silver nanoparticles, gold nanoparticles (GNPs), and nanostructures (nanoscale rods, rings, cages, crescents, or holes) are considered the most popular materials in biosensor design due to their good stability, high electrical conductivity, and unique structural and catalytic properties [10,11,12,13,14]. Dendritic gold nanostructures (DGN_S_) are considered to be one of the types of nanomaterials with great promise in electronics and biomedical applications [11,12,13,15,16,17,18,19]. The electrochemical deposition of DGNs occurs in several stages: first, the AuCl^4−^ ions are reduced to Au^0^; second, atomic gold clusters are formed with the increasing Au^0^ concentration; and third, these clusters act as seeds for DGN formation and growth [15,20,21]. The morphology of DGNs may be controlled by (i) the AuCl^4−^ ion concentration, (ii) the viscosity of the solution, (iii) the overpotential [15], and (iv) the electrodeposition duration [11]. The formation of DGNs on the electrode surface enhances the electrochemically active surface area and efficiency of electron transfer in the electrochemical detection of glucose [17]. A biosensor for the determination of glucose using a screen-printed carbon (SPC) electrode with electrochemically synthesized DGNs and GOx (SPC/DGNs/GOx) exhibited a sensitivity of 46.76 µA mM^−1^ cm^−2^ to glucose in the presence of a redox mediator [12]. A glucose biosensor based on a glassy carbon (GC) electrode modified with nanoporous gold (NPG) and GOx (GC/NPG/GOx) was characterized by a glucose sensitivity of 12.1 µA mM^−1^ cm^−2^ [13].

In enzymatic biosensors, β-D-glucose is oxidized by GOx to D-glucono-1,5-lactone, whereas oxygen is reduced to hydrogen peroxide [7,22,23]. In the presence of DGNs, the oxidation of glucose is enhanced by the synergistic effects of GOx and DGNs [13]. Inorganic, organic, or metal-organic redox mediators (Med) replace the oxygen in the reaction and transfer electrons from the reduced GOx redox center to the working electrode. Electrochemical biosensors with such mediators are characterized by a high current density due to the incensement of flavin adenine dinucleotide (FAD) and the GOx reoxidation rate during the catalytic oxidation of glucose [3,6]. Redox mediators should be characterized by a fast electrochemical reaction, reversibility, low regeneration potential, good stability, nontoxicity to the enzyme, and a lack of dependence on the solution pH [24]. Biosensors based on such redox mediators, like ferrocene derivatives (e.g., ferrocenecarboxylic acid (FCA)) [9,24,25], 1,10-phenanthroline-5,6-dione (PD) [26,27], 3,3′,5,5′-tetramethylbenzidine (TMB) [23,28], and tetrathiafulvalene (TTF) [27,29,30,31]), have been reported previously. FCA [9], PD [26], and TTF [30] accept two electrons from the GOx redox center during electrochemical reactions. Meanwhile, redox mediators in combination with nanomaterials (e.g., metallic nanoparticles, CNTs) are characterized by excellent electron transfer, stability, and high analytical response [27,28,29,32]. Usually, ferrocene derivatives are attached to gold (Au) compounds through a thiol-Au linkage [25]. It was reported that a glucose biosensor based on a GC electrode modified with NPG, cysteamine (Cys), glutaraldehyde (GA), and GOx showed a sensitivity to glucose of 1.35 µA mM^−1^ cm^−2^ in the presence of FCA (GC/NPG/Cys/GA/GOx) [17]. Another kind of mediator, PD, is used for the complexation of various transition metal ions [33]. In electrochemical biosensors, at a neutral pH value, quinone (PD(ox)) is first reduced to hydroquinone (H_2_PD(red)), and then H_2_PD(red) is reoxidized on the working electrode surface [26,32]. Nanomaterials are able to provide more space for TMB adsorption by increasing the electron density and electron transfer [28]. TMB or TTF undergoes two one-electron oxidation steps to form TMB ^+^ and TMB^2+^ (at potentials of +0.8 and +1.0 V vs. Pt/Hg/Hg_2_Cl_2(sat. KCl)_) [34] or TTF^+^ and TTF^2 +^ (+0.36 and +0.71 V vs. Pt/Hg/Hg_2_Cl_2(sat. KCl)_) [35].

Conducting polymers, e.g., polypyrrole (Ppy), have attracted great interest in clinical and environmental practices, electrocatalysis, and biomolecule immobilization due to their efficient transfer of electric charge and flexibility [36,37]. Electrochemical [38,39,40] and enzymatic [27] polymerizations can be used to form the Ppy layer in glucose biosensors. Enzymatic Ppy formation on the electrode surface modified with GOx is initiated by H_2_O_2_, which is formed during the enzymatic GOx reaction, by generating radical cations of pyrrole [41,42]. The radical cations combine to first form oligomers and then polymers [42]. A conductive Ppy film can be deposited on the surface of the electrode, providing a stable and porous matrix for the incorporation of nanomaterials and immobilization of enzymes (e.g., horseradish peroxidase (HRP), GOx, or laccase) [27,36,39]. The formed linear or branched polymers with broad molecular weight distributions are useful for biomedical and biosensing applications [36].

The aim of the present study was to select a suitable redox mediator for the development of reagentless glucose biosensors based on DGNs, to investigate the impact of the polypyrrole layer on the performance of the developed biosensors, and to test an analytical system for the determination of glucose in the serum.

## 2. Materials and Methods

### 2.1. Materials

Carbohydrates-D-(+)-glucose, D(+)-saccharose, D(+)-xylose, D(+)-galactose, D(+)-mannose, D(-)-fructose, and an enzyme—glucose oxidase (type VII, from *Aspergillus niger*, 208 units mg^−1^ protein)—were purchased from Carl Roth GmbH + Co.KG (Karlsruhe, Germany) and Fluka (Buchs, Switzerland), respectively. The solution of sodium acetate (SA) buffer (0.05 mol L^−1^ sodium acetate trihydrate (CH_3_COONa·3H_2_O) with 0.1 mol L^−1^ potassium chloride (KCl) was prepared by mixing CH_3_COONa·3H_2_O from Reanal (Budapest, Hungary) and KCl from Lachema (Neratovice, Czech Republic). Alfa alumina powder (Al_2_O_3_, 0.3 µm, type N) was purchased from Electron Microscopy Sciences (Hatfield, MA, USA). Sodium and potassium hydroxides and sulfuric acid (H_2_SO_4_) were purchased from Merck KGaA (Darmstadt, Germany) and Reanal (Budapest, Hungary); potassium nitrate (KNO_3_) and pyrrole were obtained from Acros Organics (New Jersey, NJ, USA); ferrocenecarboxylic acid was obtained from Alfa Aesar GmbH&Co KG (Karlsruhe, Germany); and hexaammineruthenium (III) chloride (Ru(NH_3_)_6_Cl_3_) was obtained from Fisher Scientific (Waltham, MA, USA). Tetrachloroauric acid trihydrate (HAuCl_4_·3H_2_O), the graphite rod (GR, diameter of 3 mm), hydrochloric acid, 1,10-phenathroline-5,6-dione, N,N,N′,N′-tetramethylbenzidine, and tetrathiafulvalene were obtained from Sigma-Aldrich (Saint Louis, MO, USA); 25% glutaraldehyde solution was obtained from Fluka Chemie GmbH (Buchs, Switzerland); and L-ascorbic acid (AA) and uric acid (UA) was obtained from AppliChem GmbH (Darmstadt, Germany). Redox mediators were dissolved in 96% ethanol received from Vilniaus degtinė (Vilnius, Lithuania). The pyrrole was filtered before the investigations through a 5 cm column filled with Al_2_O_3_ powder.

### 2.2. The Preparation of the Graphite Rod Electrode

**Figure 1:**
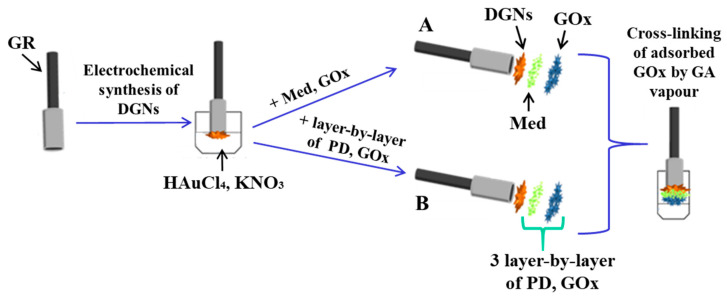
Schematic representation of the preparation of the GR/DGNs/FCA/GOx, GR/DGNs/PD/GOx, GR/DGNs/TMB/GOx or GR/DGNs/TTF/GOx (**A**), and GR/DGNs/(PD/GOx)_3_ (**B**) electrodes.

The GR electrode sealed in a silicone tube (0.071 cm^2^ of the area) was covered with long, thin, and branched DGNs, according to a previously presented methodology [18,19]. Electrochemical synthesis of the DGNs was performed with a solution of 6 mmol L^−1^ HAuCl_4_ with 0.1 mol L^−1^ KNO_3_ using a computerized potentiostat/galvanostat Autolab/PGSTAT 302N (EcoChemie, Utrecht, The Netherlands) with GPES 4.9 software at a constant potential of −0.4 V vs. Ag/AgCl_(3 mol L_^−1^_KCl)_ for 400 s [18]. Then, 4.5 µL of redox mediator (38 mmol L^−1^ FCA, PD, and TMB or 72 mmol L^−1^ TTF) was added onto the GR/DGN electrode, and the solvent was evaporated at +20 ± 2 °C. After that, the GOx from 3 µL of 25 mg mL^−1^ solution was immobilized on the GR/DGNs/Med electrode, and the solvent was evaporated at +20 ± 2 °C. Then, the modified GR electrode was stored in a closed vessel in a 25% solution of GA for 15 min at +20 ± 2 °C (Figure S1). A schematic representation of the preparation of the GR/DGNs/FCA/GOx, GR/DGNs/PD/GOx, GR/DGNs/TMB/GOx, and GR/DGNs/TTF/GOx is presented in Figure 1A. The GR/DGNs/(PD/GOx)_3_ electrode was prepared according to the described procedure using the layer-by-layer technique: PD and GOx were deposited three times onto the GR surface modified with dendritic gold nanostructures (Figure 1B).

### 2.3. Electrochemical Characterization and Evaluation of the Developed Glucose Biosensors

Current responses were registered using a computerized potentiostat/galvanostat Autolab/PGSTAT 302N with GPES 4.9 software. Electrochemical investigations were performed in unstirred or stirred (1200 rpm) 0.05 mol L^−1^ SA buffer solution, pH 6.0, via cyclic voltammetry (CV) or constant-potential amperometry (CPA). Measurements were applied using working electrodes, GR/DGNs/FCA/GOx, GR/DGNs/PD/GOx, GR/DGNs/(PD/GOx)_3_, GR/DGNs/TMB/GOx, or GR/DGNs/TTF/GOx; a 2 cm^2^ platinum spiral as an auxiliary electrode; and a Ag/AgCl_(3 mol L_^−1^_KCl)_ Metrhom (Herisau, Switzerland) as a reference electrode. Potential scans were conducted from −0.6 to +0.9 V vs. Ag/AgCl_(3 mol L_^−1^_KCl)_ for the GR/DGNs/FCA/GOx, GR/DGNs/PD/GOx, GR/DGNs/(PD/GOx)_3_, GR/DGNs/TMB/GOx, GR/DGNs/TTF/GOx, and GR electrodes and from −0.6 to +0.6 V for the GR, GR/GOx, GR/DGNs, GR/DGNs/GOx, GR/DGNs/PD, GR/DGNs/PD/GOx, and GR/DGNs/(PD/GOx)_3_ electrodes, with a 2.5 mV step potential, and a 0.05 V s^−1^ scan rate was used for the CV measurements. The CPA mode and an applied potential of +0.30 V vs. Ag/AgCl_(3 mol L_^−1^_KCl)_ were used to register the current responses using the GR/DGNs/GOx electrode; +0.40 V vs. Ag/AgCl_(3 mol L_^−1^_KCl)_ using the GR/DGNs/PD/GOx, GR/DGNs/(PD/GOx)_3_, or GR/DGNs/TTF/GOx electrodes; +0.70 V vs. Ag/AgCl_(3 mol L_^−1^_KCl)_ using the GR/DGNs/FCA/GOx electrode; and +0.75 V vs. Ag/AgCl_(3 mol L_^−1^_KCl)_ for the GR/DGNs/TMB/GOx electrode.

To determine the electroactive surface area (EASA) of the electrodeposited DGNs, cyclic voltammograms were recorded in an aqueous 0.5 mol L^−1^ H_2_SO_4_ solution in the range of 0.0 to +1.4 V. The scan rate was 0.025 V s^−1^. The EASA was estimated using the following equation [43]:

(1) $$EASA=\frac{A}{v\cdot 386\;µC\;{cm}^{-1}},$$

where *A*—the integrated peak of gold oxide reduction, *v*—the potential scan rate (V s^−1^), and 386 µC cm^−1^—the charge density per unit area associated with the electrochemical reduction of a monolayer of chemisorbed oxygen on polycrystalline gold.

To determine the EASA of the modified graphite rod electrodes, the potential was swept in the range of −0.70 to 0.0 V with varying scan rates (0.025, 0.050, 0.075, 0.100, 0.125, and 0.150 V s^−1^). The cyclic voltammograms were registered in a solution consisting of 0.1 mol L^−1^ KCl and 1 mmol L^−1^ Ru(NH_3_)_6_Cl_3_. The EASA was calculated for the GR/DGNs/TTF/GOx, GR/DGNs/(PD/GOx)_3_, GR/DGNs/TTF/GOx/Ppy_(3.5 h)_, and GR/DGNs/(PD/GOx)_3_/Ppy_(5 h)_ electrodes using the Randles–Sevcik equation:

(2) $$i_{p}=2.69\times 10^{5}\cdot n^{\frac{3}{2}}\cdot EASA\;\cdot D^{\frac{1}{2}}\;\cdot C\;\cdot v^{\frac{1}{2}},$$

where *i_p_*—maximum peak current (A), *n*—the number of electrons appearing in the half-reaction for the redox pair, *D*—diffusion coefficient (cm^2^ s^−1^), *C*—concentration of electroactive species (mol·cm^−3^), and *υ*—scan rate (V s^−1^).

All electrochemical measurements were repeated at least three times and evaluated as the mean value. The statistic software SigmaPlot (version 12.5) was used to estimate the intercept, slope, and correlation coefficient of the calibration curve, the limit of detection (LOD), the difference in maximal current response registered during the enzymatic reaction (Δ*I*_max_), and the apparent Michaelis constant (*K*_M(app)_). Δ*I*_max_ and *K*_M(app)_ were calculated as the *a* and *b* parameters of the hyperbolic function *y* = *ax*/(*b* + *x*) and were used for the approximation of the received results. The value of the LOD was estimated statistically with the software SigmaPlot 12.5 and defined as the lowest amount of glucose which provided a current response greater than the background current response value plus 3 σ.

### 2.4. The Enzymatic Synthesis of Polypyrrole and the Stability of the Developed Glucose Biosensors

To improve the analytical characteristics of the developed glucose biosensors, the enzymatic polymerization of pyrrole was performed by immersing the GR/DGNs/TTF/GOx or GR/DGNs/(PD/GOx)_3_ electrodes in a polymerization solution in the dark for 1.8, 3.5, 8, and 24 or 2, 5, 17, 21, and 46 h at +20 ± 2 °C. The polymerization solution contained 0.05 mol L^−1^ of SA buffer at pH 6.0, 0.2 mol L^−1^ of pyrrole, and 0.05 mol L^−1^ of glucose. Glucose oxidase, glucose, and oxygen are required for enzymatic polymerization to proceed [36].

The stability of the developed glucose biosensors was evaluated after the storage of the unmodified and polypyrrole-layer-modified GR/DGNs/TTF/GOx, GR/DGNs/TTF/GOx/Ppy_(3.5 h)_, GR/DGNs/(PD/GOx)_3_, and GR/DGNs/(PD/GOx)_3_/Ppy_(5 h)_ electrodes in a 0.05 mol L^−1^ SA buffer solution of pH 6.0 at +4 °C for up to 25 days.

### 2.5. The Application of a Biosensor Based on a GR/DGNs/(PD/GOx)_3_/Ppy_(5 h)_ Electrode for Glucose Detection in Serum

The serum was diluted 10 times and centrifuged (8 min, 14,600× *g*) using an IEC CL31R Multispeed centrifuge (Aze Bellitourne, Château-Gontier, France) according to the previously described method [19]. CPA investigations were performed for a glucose biosensor based on the GR/DGN/(PD/GOx)_3_/Ppy_(5 h)_ electrode. The developed glucose biosensor was tested in serum diluted 10 times with 10.0 mmol L^−1^ of glucose before and after the addition of 1.0 mmol L^−1^ of carbohydrate (saccharose, xylose, galactose, mannose, or fructose). This study involved an evaluation of the influence of electroactive species on the current response. Experiments were performed in serum diluted 10 times with either 10.0 mmol L^−1^ of glucose alone or 10.0 mmol L^−1^ of glucose with 0.01 or 0.1 mmol L^−1^ of AA or with 0.01 or 0.05 mmol L^−1^ of UA.

## 3. Results and Discussion

### 3.1. Electrochemical Characterization of the Developed Biosensors

The nanostructurization of the electrode surface is one of the methods for improving the analytical parameters of the developed biosensors. In this work, electrodeposited gold nanostructures were used for this purpose. DGNs were synthesized according to a previously published study [18,19]. DGNs have a high surface area, which increases the analytical value of their signals. The EASA of the DGNs was evaluated using Equation (1) (Figure S2). The EASA of the synthesized gold nanostructures was found to be 1.23 ± 0.01cm^2^, which is significantly larger than the geometric area (0.071 cm^2^) of the graphite rod electrode.

**Figure 2:**
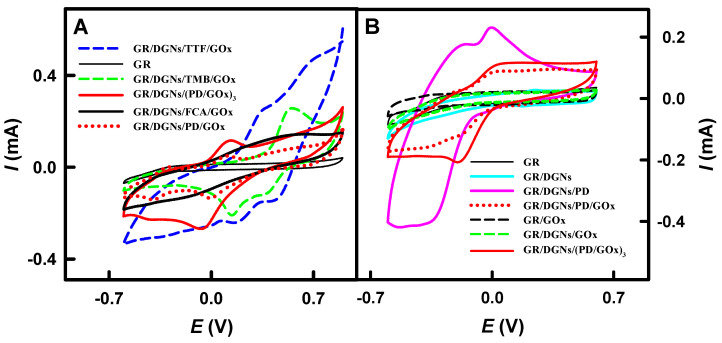
Cyclic voltammograms registered using bare GR or GR electrodes modified with DGNs, TTF, TMB, PD, or FCA (**A**) and those acquired after different stages of GR electrode modification using PD as a redox mediator (**B**). Cyclic voltammograms were recorded in 0.05 mol L^−1^ SA buffer of pH 6.0 with 0.1 mol L^−1^ KCl.

The data regarding the mechanism of the redox process and the electron transfer can be obtained in the form of a cyclic voltammogram and peak shift [37]. The electrochemical behavior of the glucose biosensors based on GR/DGNs/FCA/GOx, GR/DGNs/PD/GOx, GR/DGNs/(PD/GOx)_3_, GR/DGNs/TMB/GOx, and GR/DGNs/TTF/GOx was studied in 0.05 mol L^−1^ SA buffer of pH 6.0 with 0.1 mol L^−1^ KCl via CV according to the methodology described in Section 2.3. The registered cyclic voltammograms are presented in Figure 2A.

It is clear that electron transfer from the GOx redox center to electrodes without mediators does not take place when using the GR/GOx and GR/DGNs/GOx electrodes (Figure 2B). As can be seen from Figure 2B, electron transfer begins only in the presence of PD on the GR/DGNs electrode, and a similar behavior was observed with another redox mediator. The cyclic voltammogram registered using the GR/DGNs/FCA/GOx electrode (Figure 2A) was characterized by a small anodic peak at the potential of +0.50 V vs. Ag/AgCl_(3 mol L_^−1^_KCl)_. However, the cyclic voltammograms obtained using the GR/DGNs/PD/GOx, GR/DGNs/TMB/GOx, and GR/DGNs/TTF/GOx electrodes were characterized by clearly visible anodic and cathodic peaks (Figure 2A). Anodic peaks were noted at +0.010 V vs. Ag/AgCl_(3 mol L_^−1^_KCl)_ for the GR/DGNs/PD/GOx and GR/DGNs/(PD/GOx)_3_ electrodes and at + 0.54 V vs. Ag/AgCl_(3 mol L_^−1^_KCl)_ for the GR/DGNs/TMB/GOx electrode. The shape of the cyclic voltammograms and the position of the anodic peak for the GR/DGNs/FCA/GOx and GR/DGNs/PD/GOx electrodes are very similar to those obtained using the GC/NPG/Cys/GA/GOx electrode in the presence of FCA (+0.316 V) [17] and the GR/PD/GOx (+0.010 V) [27] electrode, respectively. The two anodic peaks at + 0.30 and +0.67 V vs. Ag/AgCl_(3 mol L_^−1^_KCl)_ were obtained in the case of the cyclic voltammograms recorded using the GR/DGNs/TTF/GOx electrode. The presence of two peaks can be explained by the reversible oxidation/reduction process between the neutral TTF, radical cation (TTF^+^), and dication (TTF^2+^) states [44]. Similar cyclic voltammograms were registered previously for the GR/TTF/GOx-electrode-based glucose biosensor, with the first oxidation peak at +0.307 V vs. Ag/AgCl_(3 mol L_^−1^_KCl)_ [27]. The obtained TTF^+^ is considered an effective redox mediator for electron transfer [31].

To ensure efficient electron transfer on the GR/DGNs/PD/GOx or GR/DGNs/TTF/GOx electrodes, the potential value of +0.40 V vs. Ag/AgCl_(3 mol L_^−1^_KCl)_ was selected for further studies, while for GR/DGNs/FCA/GOx and GR/DGNs/TMB/GOx, it was selected as +0.70 V and +0.75 V vs. Ag/AgCl_(3 mol L_^−1^_KCl)_, respectively.

### 3.2. The Selection of the Optimal Redox Mediator for Glucose Biosensor Construction

**Figure 3:**
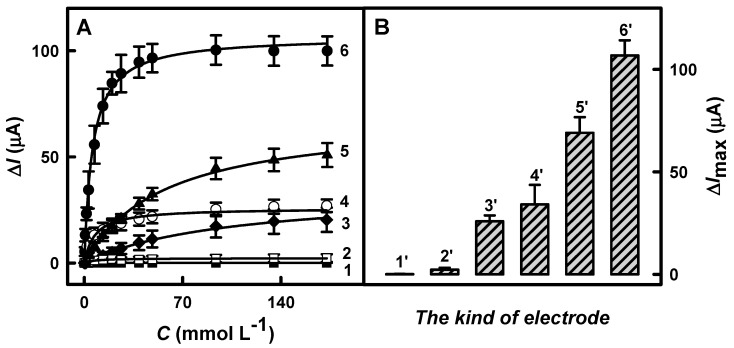
Calibration plots (**A**) and diagrams of current responses (**B**) registered using enzymatic glucose biosensors based on GR electrodes modified using DGNs without or with redox mediators. Details of the presented results: GR/DGNs/GOx (◼, 1 line, 1′ column) at +0.30 V vs. Ag/AgCl_(3 mol L_^−1^_KCl)_; GR/DGNs/FCA/GOx (◻, 2 line, 2′ column) at +0.70 V vs. Ag/AgCl_(3 mol L_^−1^_KCl)_; GR/DGNs/TMB/GOx (○, 4 line, 3′ column) at +0.75 V vs. Ag/AgCl_(3 mol L_^−1^_KCl)_; GR/DGNs/PD/GOx (◆, 3 line, 4′ column), GR/DGNs/(PD/GOx)_3_ (▲, 5 line, 5′ column), and GR/DGNs/TTF/GOx (●, 6 line, 6′ column) at +0.40 V vs. Ag/AgCl_(3 mol L_^−1^_KCl)_. Responses of CPA were registered in 0.05 mol L^−1^ SA buffer, pH 6.0, with 0.1 mol L^−1^ KCl. Typical amperograms are presented in Figure S3.

The nature of the redox mediator has a significant influence on the sensitivity of electrochemical biosensors. To evaluate the impacts of the FCA, PD, TMB, and TTF on the current responses, five types of working electrodes, including GR/DGNs/FCA/GOx, GR/DGNs/PD/GOx, GR/DGNs/(PD/GOx)_3_, GR/DGNs/TMB/GOx, and GR/DGNs/TTF/GOx, were prepared. The calibration plots using 38 mmol L^−1^ of FCA, PD, or TMB or 72 mmol L^−1^ of TTF are shown in Figure 3A. The redox mediators used were compared with each other in terms of effectiveness. It was observed that all the calibration plots were in agreement with Michaelis–Menten kinetics.

The difference in maximal current response depends on the Med used. In the case of the FCA, TMB, PD, and threefold-deposited PD redox mediators, the Δ*I*_max_ was calculated as 2.25 ± 0.92, 25.9 ± 2.8, 34.1 ± 9.6, and 69.1 ± 7.7 µA, respectively. The highest value of Δ*I*_max_ was achieved for the glucose biosensor based on the GR/DGNs/TTF/GOx electrode −107 ± 7 µA. Meanwhile, in the case of the GR/DGNs/GOx-electrode-based glucose biosensor without a Med, no current response was recorded, which was expected, since direct electron transfer is difficult in the case of GOx, because FAD, as a cofactor, is located deep in the molecule globule, which is electrically well-isolated [45].

The value of Δ*I*_max_ for the biosensor based on the GR/DGNs/(PD/GOx)_3_ electrode was 2.03 times higher than that obtained using the GR/DGNs/PD/GOx electrode. DGN fabrication leads to an increase in the electroactive surface area, and thus, an improved signal is recorded. This can be explained by the one-dimensional hollow tubular form of PD, which promotes electron transfer [46]. For further studies, biosensors based on the GR/DGNs/TTF/GOx and GR/DGNs/(PD/GOx)_3_ electrodes were selected, as the highest current responses were registered for these electrodes.

### 3.3. Influence of the Polypyrrole Layer on Biosensor Performance

**Figure 4:**
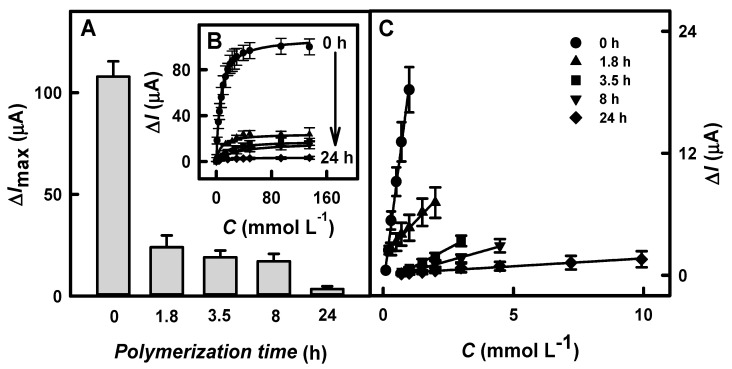
Δ*I*_max_ values (**A**), calibration plots (**B**), and linear dynamic ranges (LDR) (**C**) for biosensors based on GR/DGNs/TTF/GOx/Ppy electrodes fabricated using various polymerization times. Details of the presented results: symbols for (**B**,**C**) graphs are the same. Current responses were registered using CPA in 0.05 mol L^−1^ SA buffer, pH 6.0, with 0.1 mol L^−1^ KCl at + 0.40 V vs. Ag/AgCl_(3 mol L_^−1^_KCl)_. Typical amperograms are presented in Figure S4A.

**Figure 5:**
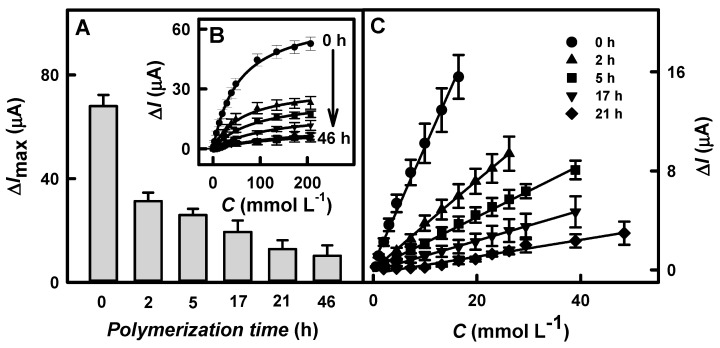
Δ*I*_max_ values (**A**), calibration plots (**B**), and linear dynamic ranges (**C**) for biosensors based on GR/DGNs/(PD/GOx)_3_/Ppy electrodes fabricated using various polymerization times. Details of the presented results: symbols for (**B**,**C**) graphs are the same. Current responses were registered using CPA in 0.05 mol L^−1^ SA buffer, pH 6.0, with 0.1 mol L^−1^ KCl at +0.40 V vs. Ag/AgCl_(3 mol L_^−1^_KCl)_. Typical amperograms are presented in Figure S4B.

To improve the performance of the biosensors based on the GR/DGNs/TTF/GOx and GR/DGNs/(PD/GOx)_3_ electrodes, the enzymatic formation of the Ppy layer was performed. Firstly, the Ppy layer was enzymatically synthesized on the surfaces of the GR/DGNs/TTF/GOx or GR/DGNs/(PD/GOx)_3_ electrodes according to the protocol described in Section 2.4. Then, the dependences of the current responses on the glucose concentration after various polymerization times were registered (Figure 4B and Figure 5B, respectively), and the Δ*I*_max_ and *K*_M(app)_ were evaluated.

Based on hyperbolic dependences, the calculated Δ*I*_max_ values for the glucose biosensors based on the GR/DGNs/TTF/GOx or GR/DGNs/(PD/GOx)_3_ electrodes after the Ppy layer’s formation depended on the Ppy layer formation time. As can be seen from Figure 4A and Figure 5A, as the polymerization time increased, a decrease in Δ*I*_max_ was observed using both of the electrodes tested. In the case of the electrode prepared using TTF, when the Ppy was synthesized for 1.8, 3.5, 8, or 24 h of enzymatic polymerization (Figure 4A), the Δ*I*_max_ decreased from 108 ± 7.5 µA (calculated for the GR/DGNs/TTF/GOx electrode) by 4.48 (Δ*I*_max_ = 24.1 ± 5.8 μA), 5.68 (Δ*I*_max_ = 19.0 ± 3.4 μA), 6.32 (Δ*I*_max_ = 17.1 ± 3.7 μA), and 31.1 (Δ*I*_max_ = 3.47 ± 1.32μA) times, respectively. In the case of the GR/DGNs/(PD/GOx)_3_-electrode-based biosensor modified with the Ppy layer during 2, 5, 17, 21, or 46 h of polymerization, the Δ*I*_max_ decreased from 68.0 ± 4.3 µA (calculated for the GR/DGNs/(PD/GOx)_3_ electrode) by 2.17 (Δ*I*_max_ = 31.3 ± 3.3), 2.63 (Δ*I*_max_ = 25.9 ± 2.4), 3.51 (Δ*I*_max_ = 19.4 ± 4.5), 5.31 (Δ*I*_max_ = 12.8 ± 3.4), and 6.67 (Δ*I*_max_ = 10.2 ± 4.0 µA) times, respectively (Figure 5A). Comparing the obtained results for the glucose biosensors based on the GR/DGNs/TTF/GOx and GR/DGNs/(PD/GOx)_3_ electrodes, we noticed that the enzymatic formation of Ppy significantly affected the performance of the tested electrodes. However, a stronger decrease in Δ*I*_max_ was observed using TTF. This could be attributed to the poor stability of the biosensor modified with TTF [29,35].

A high *K*_M(app)_ value is considered to be a significant indicator of the linear dynamic range (LDR) width of the glucose biosensor. The value of *K*_M(app)_, which was determined for the GR/DGNs/TTF/GOx electrode without Ppy and after 1.8, 3.5, and 8 h polymerization, increased from 5.97 to 6.86, 20.2, and 30.8 mmol L^−1^. The biosensors based on the unmodified GR/DGNs/(PD/GOx)_3_ electrodes and those modified with a Ppy layer for 2, 5, 17, and 21 h also showed increases in *K*_M(app)_ from 55.5 to 61.0, 91.3, 132, and 200 mmol L^−1^. Although the Ppy layer on the surface of the immobilized GR electrodes reduced the current response and the sensitivity of the developed biosensors, the high value of *K*_M(app)_ demonstrates the superiority of the polymerized electrodes over the unpolymerized ones in the practical determination of glucose in real samples, where a wide LDR is considered one of the main advantages.

According to the hyperbolic dependences shown in Figure 4B and Figure 5B, the LDR for the fabricated glucose biosensors was evaluated. As seen in Figure 4C and Figure 5C, the LDRs were without an intercept on the *x*- or *y*-axis and could be used for glucose determination. The LDRs of the GR/DGNs/TTF/GOx and GR/DGNs/(PD/GOx)_3_ electrodes were extended by increasing the duration of Ppy synthesis and depended on the kind of redox mediator used. In the case of the GR/DGNs/TTF/GOx electrodes (Figure 4C) modified with a Ppy layer for 1.8, 3.5, 8, and 24 h of polymerization, the LDRs were extended from 1 mmol L^−1^ to 2.00, 2.99, 4.48, and 9.92 mmol L^−1^ of glucose. As seen in Figure 5C, the LDRs after 2 and 5 h of Ppy layer formation on the GR/DGNs/(PD/GOx)_3_ electrode were extended from 16.5 mmol L^−1^ to 26.2 mmol L^−1^ and 39.0 mmol L^−1^ of glucose; thus, the LDR was extended by 1.59 and 2.36 times, respectively. When the polymerization time was increased to 17 h, there was no extension of the LDR. A further increase in the polymerization duration to 24 h prolonged the LDR but was accompanied by a significant decrease in the recorded current response. Taking into account the discussed analytical parameters, the GR/DGNs/TTF/GOx/Ppy_(3.5 h)_ or GR/DGNs/(PD/GOx)_3_/Ppy_(5 h)_ electrodes were chosen for further comparison and can be recommended as more suitable for glucose biosensor construction and the determination of glucose. The LDRs for the unmodified and selected electrodes were characterized by a correlation coefficient above 0.9900.

The LDR for the developed reagentless biosensor based on the GR/DGNs/TTF/GOx electrode was the same as that obtained by other authors using a reagentless biosensor based on a Au electrode modified with a Cys self-assembled monolayer, TTF, GOx, and alcohol oxidase (AOx) (Au-Cys/TTF/GOx-AOx) (up to 1.0 mmol L^−1^ of glucose) [31]. Modification with the Ppy layer increased the LDR, but this increase was not enough, as compared to almost all the other biosensors in Table 1. As mentioned earlier, the LDR was much wider (up to 39.0 mmol L^−1^) when using a reagentless biosensor based on the GR/DGNs/(PD/GOx)_3_/Ppy_(5 h)_ electrode. Thus, the LDR of this electrode was at least twice as wide as that of the other electrodes (Table 1).

The sensitivity of the biosensors based on the GR/DGNs/TTF/GOx, GR/DGNs/TTF/GOx/Ppy_(3.5 h)_, GR/DGNs/(PD/GOx)_3_ and GR/DGNs/(PD/GOx)_3_/Ppy_(5 h)_ electrodes was 67.6, 11.1, 13.4, and 3.03 µA mM^−1^ cm^−2^, respectively (Table 1). Although the Ppy layer reduced the sensitivity of the reagentless biosensors by more than four times in comparison with the unmodified electrodes, the wide LDR of glucose achieved could support the application of GR/DGNs/TTF/GOx/Ppy_(3.5 h)_ or GR/DGNs/(PD/GOx)_3_/Ppy_(5 h)_ electrodes for analyte detection in real samples.

The biosensors developed based on GR/DGNs/TTF/GOx, GR/DGNs/TTF/GOx/Ppy_(3.5 h)_, GR/DGNs/(PD/GOx)_3_ and GR/DGNs/(PD/GOx)_3_/Ppy_(5 h)_ electrodes in the presence of 29.4 mmol L^−1^ glucose were characterized by good repeatability; the relative standard deviation (RSD) was evaluated as 9.65, 8.53, 9.10, and 9.03%, respectively. After the addition of glucose into the electrochemical cell, 95% of the registered current response was reached within 8 s, and it was 1.38 times faster than that achieved using the GC/OOPpy_(300 s)_-GNPs/GOx electrode (11 s) [39] and 2.5 times faster than that in our previous research, where GR electrodes modified with long-chain Ppy/GNPs_(6nm)_-GOx- or Ppy/GNPs_(AuCl4_^−^_)_-GOx nanocomposites were used (20 s) [47].

**Table 1:** Summary of the analytical characteristics commonly evaluated in glucose biosensors.

Working Electrode; Redox Mediator	LOD (mmol L^−1^)/ Sensitivity (μA mM^−1^ cm^−2^)	LDR (mmol L^−1^)	Reference
GR/DGNs/GOx; PMS in solution	0.059/169	0.1–9.97	[18]
GR/DGNs/GOx/Ppy_(22 h)_; PMS in solution	0.070/59.4	0.1–19.9	[19]
GR/GNPs_(3.5nm)_/PD/GOx	0.024/52.1	0.1–10.0	[27]
GR/PD/GOx	0.095/28.5		
SPC/DGNs/GOx; K_3_[Fe(CN_6_)] in solution	0.007/46.76	0.028–8.4	[12]
Carbon ink/GOx/HRP; K_4_[Fe(CN_6_)] in solution	0.03/−	0.05–1.0	[48]
GC/NPG/GOx; −	0.00102/12.1	0.05–10	[13]
Au-Cys/TTF/GOx-AOx	0.03/−	0.1–1.0	[31]
GC/OOPpy_(300 s)_-GNPs/GOx; −	0.5/−	1.0–8.0	[39]
GR/DGNs/TTF/GOx	0.012/67.6	0.10–1.00	This work This work This work This work
GR/DGNs/TTF/GOx/Ppy_(3.5 h)_	0.078/11.1	0.70–2.99	
GR/DGNs/(PD/GOx)_3_	0.114/13.4	0.50–16.5	
GR/DGNs/(PD/GOx)_3_/Ppy_(5 h)_	0.683/3.03	2.0–39.0	

AOx—alcohol oxidase, Cys—cysteamine, GC—glassy carbon, HRP—horseradish peroxidase, K_3_[Fe(CN_6_)]—potassium ferricyanide, K_4_[Fe(CN_6_)]—potassium ferrocyanide, NPG—nanoporous gold, OOPpy_(300 s)_—overoxidized polypyrrole, PMS—phenazine methosulfate, SPC—screen-printed carbon.

The type of redox mediator used and the polymerization duration affected the LOD of the developed reagentless biosensors. The LODs for the glucose biosensors based on GR/DGNs/TTF/GOx or GR/DGNs/TTF/GOx/Ppy_(3.5 h)_ electrodes were 9.50 and 8.76 times lower than that obtained using GR/DGNs/(PD/GOx)_3_ or GR/DGNs/(PD/GOx)_3_/Ppy_(5 h)_ electrodes (Table 1). The LOD of the reagentless glucose biosensor developed in this study based on a GR/DGNs/TTF/GOx electrode was 4.92 and 2.50 times lower than that of the glucose biosensors based on the GR/DGNs/GOx electrode in the presence of PMS (0.059 mmol L^−1^) [18] and on the Au-Cys/TTF/GOx-AOx electrode (0.03 mmol L^−1^) [31], respectively. Although the low value of the LOD presents an advantage of the glucose biosensor based on the GR/DGNs/TTF/GOx vs. GR/DGNs/(PD/GOx)_3_ electrode, a wide LDR was observed using PD in the construction of the biosensors.

The electroactive surface area of the electrodes was calculated according to the methodology described in Section 2.3. Ru(NH_3_)_6_Cl_3_ was used as a redox probe, because its oxidation occurs in a region where the studied electrodes do not yield an additional signal (Figure 2 and Figure S5). For the calculation, a value of the Ru(NH_3_)_6_Cl_3_ diffusion coefficient equal to 9.1 × 10^−6^ cm^2^ s^−1^ was used [49]. The EASA of the GR/DGNs/TTF/GOx, GR/DGNs/TTF/GOx/Ppy_(3.5 h)_, GR/DGNs/(PD/GOx)_3_, and GR/DGNs/(PD/GOx)_3_/Ppy_(5 h)_ electrodes was calculated as 0.504, 0.648, 0.736, and 0.901 cm^2^, respectively, according to the results presents in Figure S6. Modifying the electrodes with the Ppy layer allowed for an EASA increase of more than 20%. The EASA of the electrodes obtained using PD was larger than that of the electrodes fabricated using TTF.

### 3.4. The Stability of Glucose Biosensors

**Figure 6:**
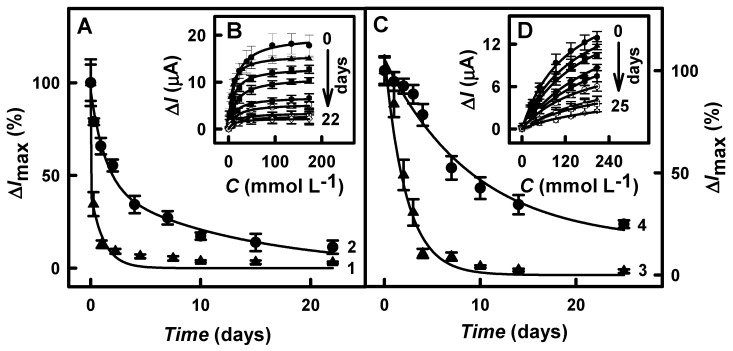
Changes in current responses over time (**A**,**C**) and calibration plots (**B**,**D**) of glucose biosensors based on unmodified and Ppy-layer-modified GR/DGNs/TTF/GOx (**A**,**B**) or GR/DGNs/(PD/GOx)_3_ electrodes (**C**,**D**). Details of the presented plots: GR/DGNs/TTF/GOx electrode (**A**—▲, line 1), GR/DGNs/TTF/GOx/Ppy_(3.5 h)_ (**A**—●, line 2, **B**—all lines), GR/DGNs/(PD/GOx)_3_ (**C**—▲, line 3), and GR/DGNs/(PD/GOx)_3_/Ppy_(5 h)_ (**C**—●, line 4, **D**—all lines). Responses of CPA were registered in 0.05 mol L^−1^ SA buffer, pH 6.0, with 0.1 mol L^−1^ KCl at + 0.40 V vs. Ag/AgCl_(3 mol L_^−1^_KCl)_. Typical amperograms for the GR/DGNs/TTF/GOx, GR/DGNs/TTF/GOx/Ppy_(3.5 h)_, GR/DGNs/(PD/GOx)_3_, and GR/DGNs/(PD/GOx)_3_/Ppy_(5 h)_ electrodes are presented in Figures S7A,B and S8A,B, respectively.

The stability of biosensors is an essential parameter that defines the duration of their use [7]. Thus, the GR/DGNs/TTF/GOx, GR/DGNs/TTF/GOx/Ppy_(3.5 h)_, GR/DGNs/(PD/GOx)_3_, and GR/DGNs/(PD/GOx)_3_/Ppy_(5 h)_ electrodes were stored at +4 °C in SA buffer solution at pH 6.0 for up to 22 and 25 days, respectively. The variation in the current responses over time using the glucose biosensors based on electrodes modified with TTF or PD is shown in Figure 6A,C, respectively. As presented in Figure 6B,D, the calibration plots were in agreement with Michaelis–Menten kinetics.

As seen in Figure 6A,C, the current responses of the glucose biosensors based on the GR/DGNs/TTF/GOx (line 1) and GR/DGNs/(PD/GOx)_3_ (line 3) electrodes significantly decreased during the first two days, and only 12.9 or 49.0% of their initial value was retained. However, the modification of the developed electrodes with the Ppy layer could increase the stability of the biosensors. The current responses obtained using the GR/DGNs/TTF/GOx/Ppy_(3.5 h)_ (Figure 6A, line 2) and GR/DGNs/(PD/GOx)_3_/Ppy_(5 h)_ (Figure 6C, line 4) electrodes were found to decrease over the studied period and retained 11.3 and 24.9% of the initial current response after 22 and 25 h, respectively. The decrease in current response could be related to GOx inactivation and Ppy degradation during the maintenance of the glucose biosensor over time. It can be seen that the biosensor based on GR/DGNs/TTF/GOx/Ppy_(3.5 h)_ retained 44.7% of the initial current response after the first two days, whereas the GR/DGNs/(PD/GOx)_3_/Ppy_(5 h)_ electrode was much more stable, as it retained 92.5% of the initial current response. These results suggest that the Ppy-modified electrodes are preferable to the unmodified ones.

The reagentless glucose biosensors based on the GR/DGNs/TTF/GOx/Ppy_(3.5 h)_ or GR/DGNs/(PD/GOx)_3_/Ppy_(5 h)_ electrodes retained 50% of their initial current response (*τ*_1/2_) after 2.0 and 9.0 days, respectively. It is obvious that the glucose biosensor based on the GR/DGNs/(PD/GOx)_3_/Ppy_(5 h)_ electrode is 4.5 times more stable than that based on the GR/DGNs/TTF/GOx/Ppy_(3.5 h)_ electrode. After *τ*_1/2_ = 9.0 days, the reagentless glucose biosensor based on the GR/DGNs/(PD/GOx)_3_/Ppy_(5 h)_ electrode was 2.73 times more stable than that obtained in our previous research using a GR/Ppy/GNPs_(6nm)_-GOx/GOx (*τ*_1/2_ = 3.3 days) electrode [47].

The noticeably, the poor TTF stability noted in a previous paper [29] and established in our studies and narrow LDR influenced the choice of the GR/DGNs/(PD/GOx)_3_/Ppy_(5 h)_ electrode for the reagentless glucose biosensor’s construction and further glucose determination in blood serum.

### 3.5. Glucose Determination in Blood Serum Using the Developed Glucose Biosensor Based on the GR/DGNs/(PD/GOx)_3_/Ppy_(5 h)_ Electrode

Saccharose, xylose, galactose, mannose, and fructose are considered as species interfering in glucose biosensing. The ascorbic acid and uric acid belong to electroactive species that can affect the current response of glucose biosensors in the blood serum [4,50,51,52].

**Figure 7:**
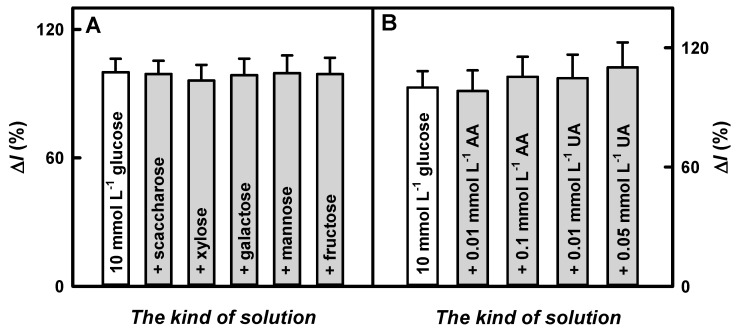
The influences of carbohydrates (1.0 mmol L^−1^) (**A**, grey color) and electroactive species (**B**, grey color) on the current responses of a biosensor based on a GR/DGNs/(PD/GOx)_3_/Ppy_(5 h)_ electrode in the presence of 10.0 mmol L^−1^ of glucose (white color). Responses of CPA were registered in 10-times-diluted serum at +0.40 V vs. Ag/AgCl_(3 mol L_^−1^_KCl)_. AA—ascorbic acid; UA—uric acid.

The selectivity of the reagentless glucose biosensor based on the GR/DGNs/(PD/GOx)_3_/Ppy_(5 h)_ electrode to interfering and electroactive species was studied in 10-times-diluted serum according to the protocol described in Section 2.5. Firstly, the selectivity of the GR/DGNs/(PD/GOx)_3_/Ppy_(5 h)_ electrode was evaluated, and it is summarized in Figure 7A. As can be seen from the obtained results, the current response registered by the biosensor based on the GR/DGNs/(PD/GOx)_3_/Ppy_(5 h)_ electrode was almost the same after the addition of similar carbohydrates, which confirms its selectivity to glucose.

The next step was to study the effect of ascorbic acid and uric acid on the current response registered in the presence of glucose. It is established that the normal physiological concentration of these electroactive species in human blood serum does not normally exceed 0.141 mmol L^−1^ of AA [53] and 0.1 mmol L^−1^ of UA [50,54]. These acids, when present in biological samples, can be oxidized at a higher positive potential than that required for glucose oxidation [14]. The addition of 10.0 mmol L^−1^ glucose with 0.01 or 0.1 mmol L^−1^ of AA increased the current response to glucose obtained the using GR/DGNs/(PD/GOx)_3_/Ppy_(5 h)_ electrode by 1.80 and 5.39%, compared with the results obtained without AA (Figure 7B). The GR/DGNs/(PD/GOx)_3_/Ppy_(5 h)_-electrode-based reagentless glucose biosensor developed in this study was 1.79 and 1.14 times more resistant to 0.01 and 0.1 mmol L^−1^ of AA than the GR/Ppy/GNPs_(AuCl4_^−^_)_-GOx/GOx electrode (the interferences of 3.23 and 6.16% for 0.01 or 0.1 mmol L^−1^ AA were registered) [47]. The addition of 10.0 mmol L^−1^ glucose containing 0.01 and 0.05 mmol L^−1^ of UA increased the current response of the GR/DGNs/(PD/GOx)_3_/Ppy_(5 h)_ electrode by 4.69 and 10.2%, respectively, compared with the results registered after adding 10.0 mmol L^−1^ of glucose without UA (Figure 7B). The current responses of the previously developed glucose biosensor based on the GR/Ppy/GNPs_(AuCl4_^−^_)_-GOx/GOx electrode changed by 2.19 and 13.4%, respectively, after the addition of 10.0 mmol L^−1^ of glucose containing 0.01 or 0.05 mmol L^−1^ of UA [47]. In general, an interference of 10% is considered acceptable for electroactive species [3,52]. The reagentless biosensor based on the GR/DGNs/(PD/GOx)_3_/Ppy_(5 h)_ electrode is characterized by good glucose selectivity and can be used for blood serum glucose analysis.

The stated normal glucose concentration in human blood is usually below 5.6 mmol L^−1^ [3,14,39,54], while in diabetic patients, it can rise to 30 mmol L^−1^ [3,39]. The suitability of the developed reagentless biosensor based on the GR/DGNs/(PD/GOx)_3_/Ppy_(5 h)_ electrode for glucose detection in serum was tested using the addition method. The results obtained from three measurements were expressed as average values and are presented in Table 2. The recovery ratios using the developed reagentless glucose biosensor were in the range of 97.5 ± 6.5 to 98.0 ± 6.3%. The developed glucose biosensor based on the GR/DGNs/(PD/GOx)_3_/Ppy_(5 h)_ electrode, according to its recovery ratio (97.5–98.0%) and relative error (4.76–6.50%), is no worse than the commercial methods and sensors. As reported by Conzales’s group, the recovery ratio and relative error depend on the country, agency, method of detection, and amount of glucose [55]. For example, in the USA, for ≥75 mg dL^−1^ (4.17 mmol L^−1^) of glucose, the detection criterion is 98 ± 15%; in Europe, for ≥100 mg dL^−1^ (5.55 mmol L^−1^) of glucose, it is −95 ± 15%; and for people with type 1 diabetes, it is −99% [55].

It should be noted that the advantages of the developed reagentless glucose biosensor based on the GR/DGNs/(PD/GOx)_3_/Ppy_(5 h)_ electrode are as follows: (i) a low LOD (0.683 mmol L^−1^), wide LDR (up to 39.0 mmol L^−1^ of glucose), and good repeatability (the RSD was 9.03%); (ii) a good storage stability (50% of the current response was retained after 9.0 days); (iii) the fast determination of glucose (approximately 8 s); and (iv) high resistance to electroactive species and suitability for glucose detection in blood serum (97.5–98.0%). The electrochemical biosensor, after some improvements and mandatory clinical validation, could be used in clinical practice for the quantitative determination of glucose.

## 4. Conclusions

In this study, we developed an enzymatic reagentless glucose biosensor based on a GR electrode modified with electrochemically synthesized DGNs in combination with redox mediators such as TTF and PD, which had the simplicity to operate at a low cost with a quick response, high current response, and good sensitivity. After the formation of the Ppy layer, the reagentless biosensor based on the GR/DGNs/(PD/GOx)_3_/Ppy_(5 h)_ electrode was characterized by a wider LDR and higher stability than that based on the GR/DGNs/TTF/GOx/Ppy_(3.5 h)_ electrode. The limitation of the GR/DGNs/(PD/GOx)_3_/Ppy_(5 h)_ electrode was its 3.03 µA mM^−1^ cm^−2^ sensitivity in comparison to GR/DGNs/(PD/GOx)_3_ electrode (13.4 µA mM^−1^ cm^−2^). However, characterized by a wide LDR (up to 39.0 mmol L^−1^) and good stability, the biosensor based on the GR/DGNs/(PD/GOx)_3_/Ppy_(5 h)_ electrode was adapted for practical application and the determination of glucose in blood serum in the presence of interfering species. This principle of biosensor development can be adapted to other types of reagentless biosensors suitable for the analysis of real samples. The developed and improved reagentless glucose biosensor, due to its wide LDR, could be applied in various research areas, e.g., for biomedical purposes, for the control of beverages, for biofuel cell fabrication, and in bioelectronics devices.

## Figures and Tables

**Table 2:** The recovery ratio of glucose in serum investigated with a biosensor based on a GR/DGNs/(PD/GOx)_3_/Ppy_(5 h)_ electrode (*n* = 3).

Added Concentration (mmol L^−1^)	Detected * Concentration (mmol L^−1^)	RSD (%)	Recovery Ratio (%)
10.0	9.78 ± 0.47	4.76	97.8
20.0	19.5 ± 1.3	6.50	97.5
25.0	24.5 ± 1.5	6.31	98.0
30.0	29.3 ± 1.6	5.51	97.7

* Responses of CPA were registered in 10-times-diluted serum at + 0.40 V vs. Ag/AgCl_(3 mol L_^−1^_KCl)_.

## Data Availability

The data presented in this study are available on request from the first author.

## Supplementary Materials

The following supporting information can be downloaded at: https://www.mdpi.com/article/10.3390/bios13070727/s1, Figure S1: Image of modified GR electrode storage in a closed vessel in a 25% solution of GA for 15 min at +20 ±2 °C; Figure S2: Cyclic voltammogram registered using a GR/DGNs electrode in 0.5 mol L^−1^ H_2_SO_4_ solution; Figure S3: Amperograms registered using enzymatic glucose biosensors based on GR electrodes modified by DGNs, without or with redox mediators. Experiments were performed in 0.05 mol L^−1^ SA buffer, pH 6.0, with 0.1 mol L^−1^ KCl; Figure S4: Amperograms registered using biosensors based on GR/DGNs/TTF/GOx/Ppy (A) and GR/DGNs/(PD/GOx)_3_/Ppy (B) electrodes fabricated using various polymerization times. Experiments were performed in 0.05 mol L^−1^ SA buffer, pH 6.0, with 0.1 mol L^−1^ KCl at + 0.40 V vs. Ag/AgCl_(3 mol L_^−1^_KCl)_; Figure S5: Cyclic voltammograms of GR/DGNs/TTF/GOx (A), GR/DGNs/TTF/GOx/Ppy_(3.5 h)_ (B), GR/DGNs/(PD/GOx)_3_ (C), and GR/DGNs/(PD/GOx)_3_/Ppy_(5 h)_ (D) electrodes registered at different scan rates ranging from 0.025 to 0.150 V s^−1^ in a solution of 0.1 mol L^−1^ KCl with 1 mmol L^−1^ Ru(NH_3_)_6_Cl_3_; Figure S6: The relationships between the square root of the scan rate and registered peak anodic current; Figure S7: Amperograms registered at different times after the preparation of biosensors based on GR/DGNs/TTF/GOx (A) and GR/DGNs/TTF/GOx/Ppy_(3.5 h)_ (B) electrodes. Experiments were performed in 0.05 mol L^−1^ SA buffer, pH 6.0, with 0.1 mol L^−1^ KCl at + 0.40 V vs. Ag/AgCl_(3 mol L_^−1^_KCl)_; Figure S8: Amperograms registered at different times after the preparation of biosensors based on GR/DGNs/(PD/GOx)_3_ (A) and GR/DGNs/(PD/GOx)_3_/Ppy_(5 h)_ (B) electrodes. Experiments were performed in 0.05 mol L^−1^ SA buffer, pH 6.0, with 0.1 mol L^−1^ KCl at + 0.40 V vs. Ag/AgCl_(3 mol L_^−1^_KCl)_.
