# Supplementary material for: The Development and Evaluation of Reagentless Glucose Biosensors Using Dendritic Gold Nanostructures as a Promising Sensing Platform
Source: Biosensors (Basel). 2023 Jul 13;13(7):727. doi: 10.3390/bios13070727 (PMC10377297; doi:10.3390/bios13070727)
Supplement: Supplementary file 1 [file biosensors-13-00727-s001.zip › biosensors-2457535-supplementary.pdf]

The Development and Evaluation of Reagentless Glucose Biosensors Using Dendritic Gold Nanostructures as a Promising Sensing Platform

Natalija German [1], Anton Popov [1,2] and Almira Ramanaviciene [1,2,]*

[1] Department of Immunology, State Research Institute Centre for Innovative Medicine, LT-08406, Vilnius, Lithuania; natalija.german@imcentras.lt (N.G.); anton.popov@chgf.vu.lt (A.P.)
[2] NanoTechnas – Center of Nanotechnology and Materials Science, Faculty of Chemistry and Geosciences, Vilnius University, LT-03225, Vilnius, Lithuania
* Correspondence: almira.ramanaviciene@chf.vu.lt

Supplementary data

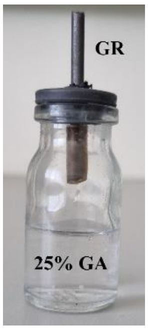

Figure S1. Image of GOx modified GR electrode storage in a closed vessel over 25% solution of GA for 15 min at +20 ± 2 °C.

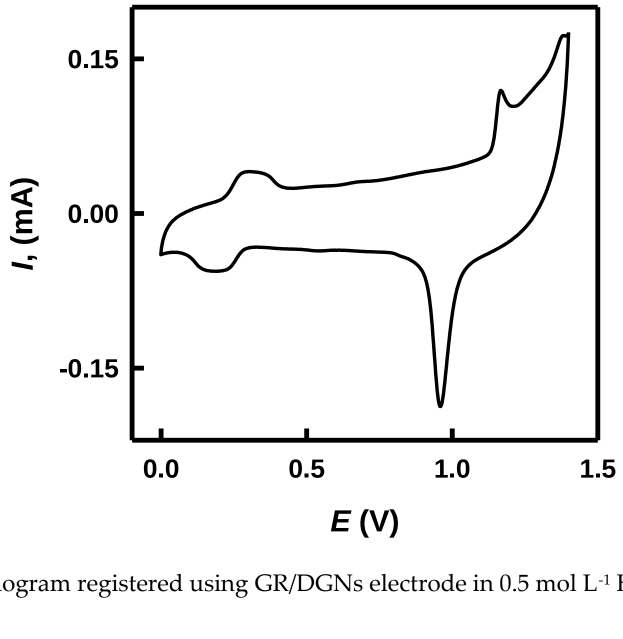

Figure S2. Cyclic voltammogram registered using GR/DGNs electrode in 0.5 mol L^{-1} H_2SO_4 solution.

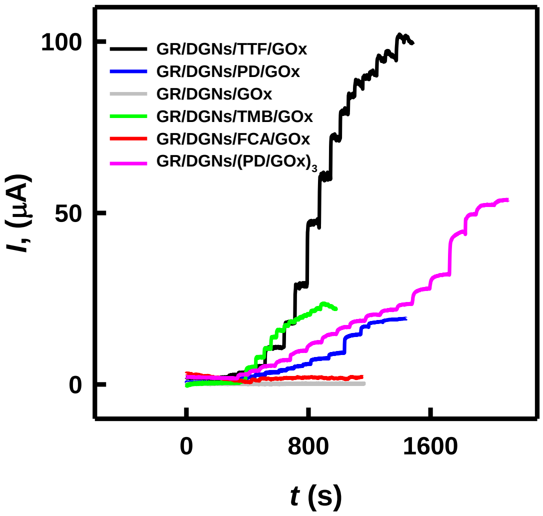

Figure S3. Amperograms registered using enzymatic glucose biosensors based on GR electrodes modified by DGNs, without or with redox mediators. Experiments were performed in 0.05 mol L^{-1} SA buffer, pH 6.0, with 0.1 mol L^{-1} KCl. (Supplement to Figure 3)

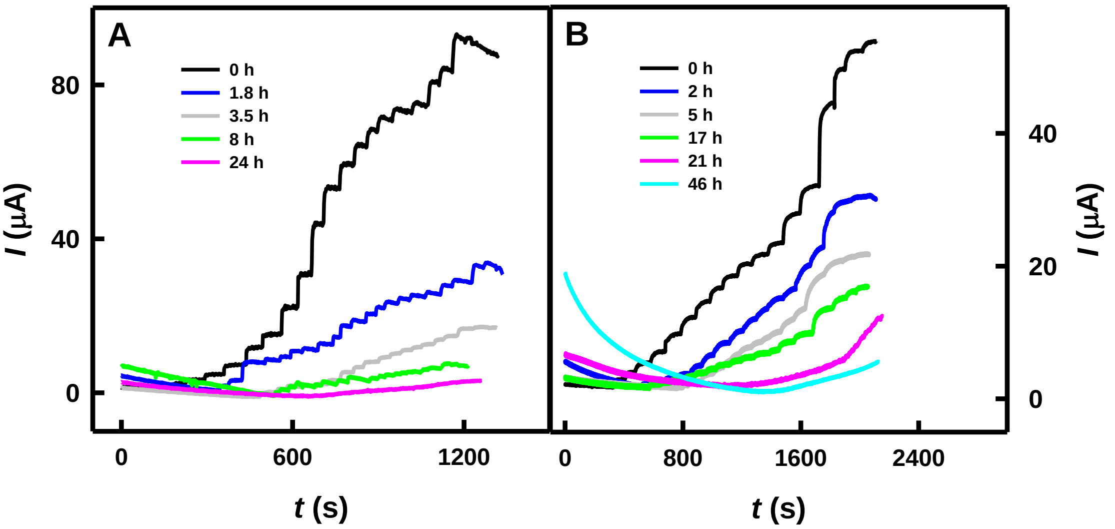

Figure S4. Amperograms registered using biosensors based on GR/DGNs/TTF/GOx/Ppy (**A**) and GR/DGNs/(PD/GOx)3/Ppy (**B**) electrodes fabricated using various polymerization times. Experiments were performed in 0.05 mol L^{-1} SA buffer, pH 6.0, with 0.1 mol L^{-1} KCl at +0.40 V vs Ag/AgCl$_{(3\ mol\ L^{-1}\ KCl)}$. (Supplement to Figures 4 and 5)

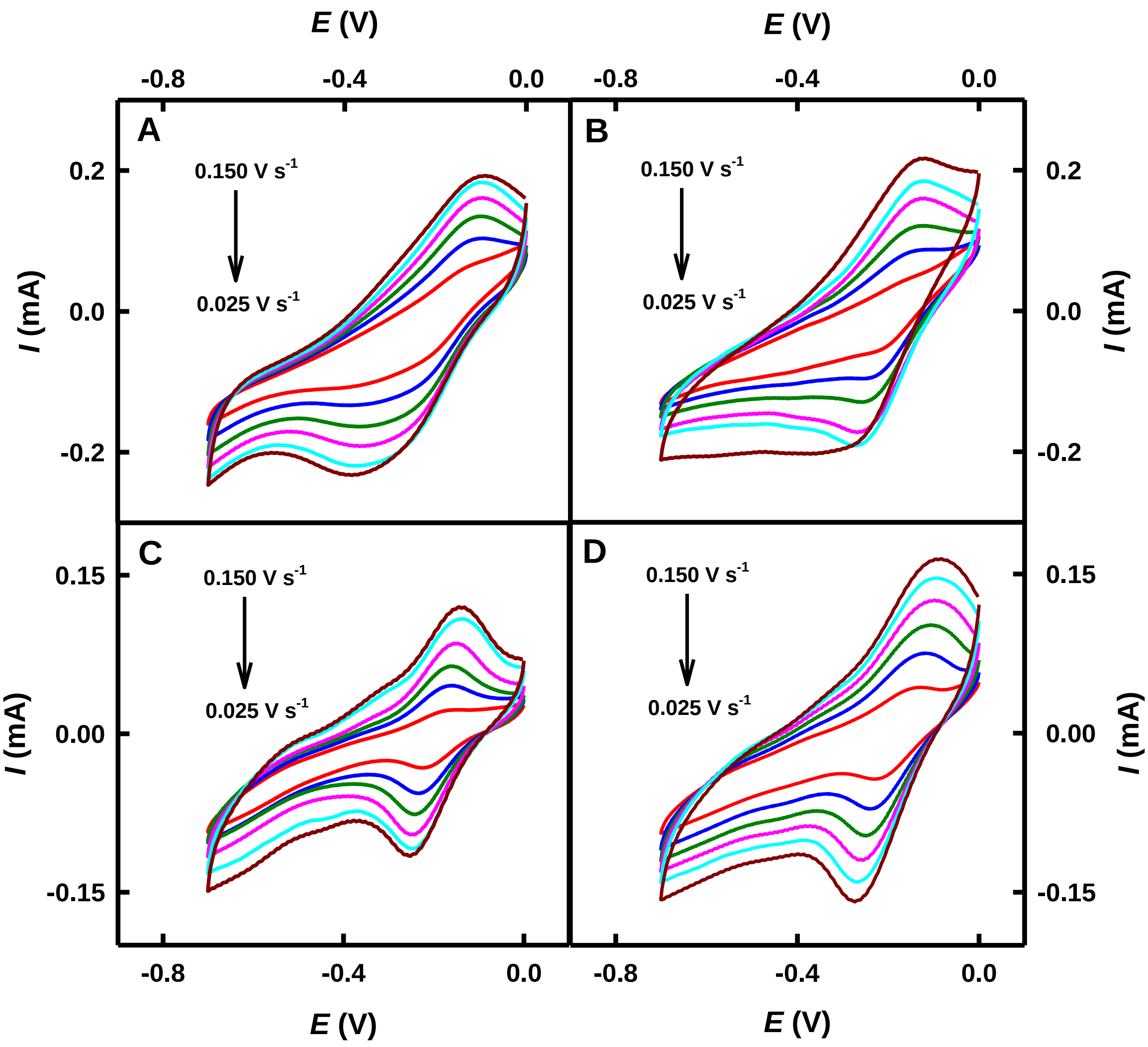

Figure S5. Cyclic voltammograms of GR/DGNs/TTF/GOx (**A**), GR/DGNs/TTF/GOx/Ppy(3.5 h) (**B**), GR/DGNs/(PD/GOx)3 (**C**) and GR/DGNs/(PD/GOx)3/Ppy(5 h) (**D**) electrodes registered at different scan rates from 0.025 to 0.150 V s^{-1} in the solution of 0.1 mol L^{-1} KCl with 1 mmol L^{-1} $Ru(NH_3)_6Cl_3$.

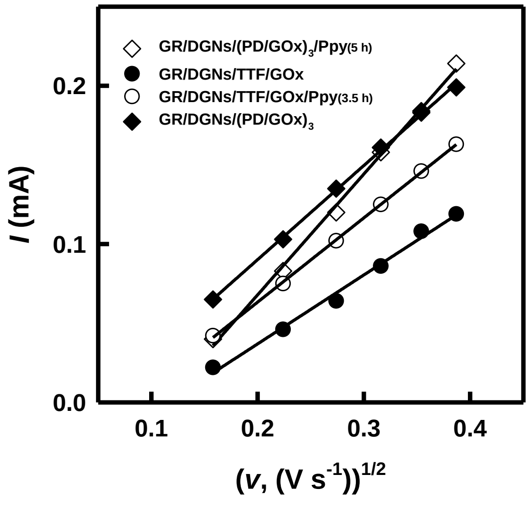

Figure S6. The relationships between square root of scan rate and registered peak anodic current.

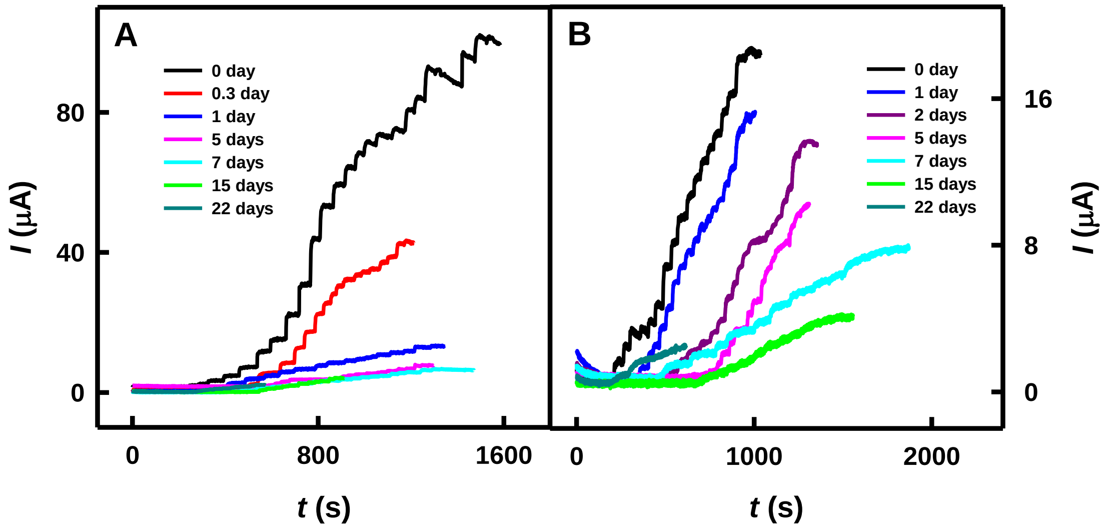

Figure S7. Amperograms registered at different times after preparation of biosensors based on GR/DGNs/TTF/GOx (**A**) and GR/DGNs/TTF/GOx/Ppy$_{(3.5\ h)}$ (**B**) electrodes. Experiments were performed in 0.05 mol L^{-1} SA buffer, pH 6.0, with 0.1 mol L^{-1} KCl at +0.40 V vs Ag/AgCl$_{(3\ mol\ L^{-1}\ KCl)}$. (Supplement to Figure 6A,B)

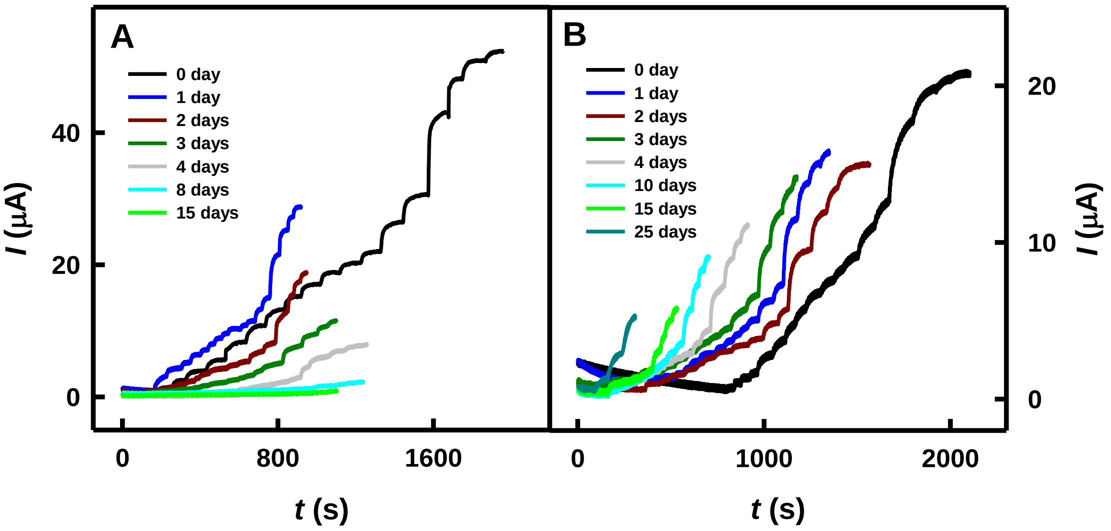

Figure S8. Amperograms registered at different times after preparation of biosensors based on GR/DGNs/(PD/GOx)$_3$ (**A**) and GR/DGNs/(PD/GOx)$_3$/Ppy$_{(5\ h)}$ (**B**) electrodes. Experiments were performed in 0.05 mol L^{-1} SA buffer, pH 6.0, with 0.1 mol L^{-1} KCl at +0.40 V vs Ag/AgCl$_{(3\ mol\ L^{-1}\ KCl)}$. (Supplement to Figure 6C,D)
